# Pedicle Morphometry Variations in Individuals with Degenerative Lumbar Spinal Stenosis

**DOI:** 10.1155/2020/7125914

**Published:** 2020-02-21

**Authors:** Janan Abbas, Natan Peled, Israel Hershkovitz, Kamal Hamoud

**Affiliations:** ^1^Department of Anatomy and Anthropology, Sackler Faculty of Medicine, Tel Aviv University, Tel Aviv 6997801, Israel; ^2^Department of Physical Therapy, Zefat Academic College, Zefat 13206, Israel; ^3^Department of Radiology, Carmel Medical Center, Haifa 3436212, Israel; ^4^Azrieli Faculty of Medicine, Bar-Ilan University, Safed 1311502, Israel; ^5^Department of Orthopaedic Surgery, The Baruch Padeh Poriya Medical Center, Tiberias 1520800, Israel

## Abstract

The aim of this study was to compare pedicle dimensions in degenerative lumbar spinal stenosis (DLSS) with those in the general population. A retrospective computerized tomography (CT) study for lumbar vertebrae (L1 to L5) from two sample populations was used. The first included 165 participants with symptomatic DLSS (age range: 40-88 years, sex ratio: 80 M/85 F), and the second had 180 individuals from the general population (age range: 40-99 years, sex ratio: 90 M/90 F). Both males and females in the stenosis group manifested significantly greater pedicle width than the control group at all lumbar levels (*P* < 0.05). In addition, pedicle heights for stenosis females were remarkably smaller on L4 and L5 levels compared to their counterparts in the control group (*P* < 0.001). Males have larger pedicles than females for all lumbar levels (*P* < 0.001). Age and BMI did not demonstrate significant association with pedicle dimensions. Our outcomes indicate that individuals with DLSS have larger pedicle widths than the control group. More so, pedicle dimensions are gender-dependent but independent of age and BMI.

## 1. Introduction

Degenerative lumbar spinal stenosis (DLSS) is a common condition in the elderly population that is related to degeneration of the three-joint complex and ligaments [1, 2]. Symptomatic DLSS requires the combination of clinical presentation with radiological findings such as computerized tomography (CT) scan and magnetic resonance imaging (MRI) modalities [3, 4]. Typically, neurogenic claudication and radicular pain are the best described clinical pictures [3].

The surgical treatment of symptomatic DLSS often requires the use of instrumentation systems. The transpedicular screw fixation is stable and worthwhile as it provides three-dimensional fixation and is increasingly used worldwide [5, 6]. It has been found that the pedicle bone is the strongest part of the vertebra, even in an osteoporotic one [7, 8]. Information regarding pedicle morphometry is essential for using the pedicle screws.

Many previous studies concerning pedicle morphometry have been conducted in subjects of various ethnic origins and populations (e.g., Western and Asian) in order to establish normal range parameters [5, 9–19]. All of these studies based their measurements on various approaches such as direct cadavers [5, 9, 11, 12, 16, 19], radiologic techniques [10, 15, 17, 18], and combined cadavers and radiologic methods [13, 14]. It has been stated that CT measurement is the best means of evaluating pedicle radiographic morphology [18, 20, 21]. Likewise, others have shown that data obtained from a CT scan for pedicle parameters are almost identical to those obtained from direct cadaveric measurements [7, 13, 22].

Because a previous study has found that lumbar vertebral bodies for DLSS individuals are significantly greater compared to those found in the general population [23], we hypothesized that the pedicle morphology for the DLSS population will be varied.

The aim of this study was to assess the pedicle parameters for the DLSS population and to compare these parameters with those for the general population.

## 2. Materials and Methods

### 2.1. Study Design

This is a cross-sectional retrospective study with two groups of individuals [24]. The first group (control) included 180 individuals without spinal stenosis-related symptoms (age range: 40-99 years, sex ratio: 90 M/90 F) who were referred to the Department of Radiology, Carmel Medical Center, Haifa, Israel, for abdominal CT scans due to abdominal problems. The second group included 165 patients with symptomatic DLSS (age range: 40-88 years, sex ratio: 80 M/85 F), who had intermittent claudication accompanied by other symptoms related to spinal stenosis [25]. Their CT scan images showed a reduced cross-sectional area (CSA) of the dural sac (<100 mm^2^) [26] of at least one lumbar level. The diagnostic criteria for DLSS were based on the combination of symptoms and signs together with the imaging findings [3]. Individuals under 40 years of age as well as those with congenital stenosis (AP diameter of the bony canal < 12 mm) [27], fractures, spondylolysis, tumors, Paget's disease, steroid treatment, severe lumbar scoliosis (>20 degrees), and iatrogenic conditions (postlaminectomy, postfusion) were excluded from the study.

A high-resolution CT image (Brilliance 64, Philips Medical Systems; voltage 120 kV, current 150–570 mA) was utilized which enabled scan processing in all planes. All CT images for both groups were taken in the same position.

This research was approved by the ethical committee of the Carmel Medical Center (0083-07-CMC).

### 2.2. Pedicle Dimensions

#### 2.2.1. Pedicle Width (PW)/Transverse Pedicle Parameter

**Figure 1:**
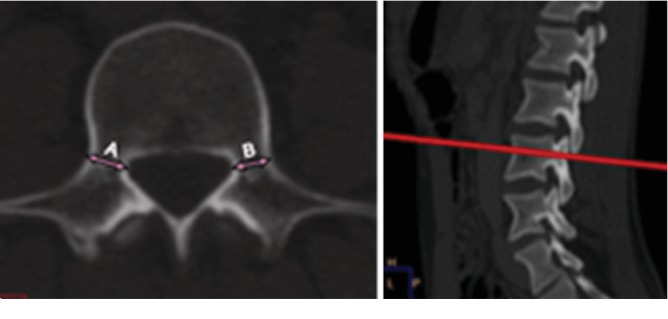
Measurement of pedicle width as conducted on an axial CT scan (a) at the middle height of the pedicle (b).

This parameter was measured in the axial plane at the middle of the pedicular height and defined as the distance between its medial and lateral cortices (Figure 1). The measurements were done on both sides (left and right), and the mean values were then calculated.

#### 2.2.2. Pedicle Height (PH)

**Figure 2:**
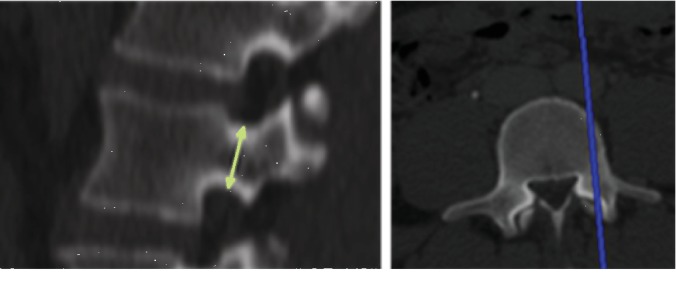
Measurement of pedicle height as conducted on a sagittal CT scan (a) at the middle of pedicle width (b).

This parameter was measured in the sagittal plane at the middle of the pedicle width and was defined as the distance between its superior and inferior cortices (Figure 2). The measurements were taken on both sides (left and right), and the averages were then calculated.

In order to identify the association between pedicle diameters and age, we classified the control group into two age groups: (a) the middle group included individuals between 40 and 60 years and (b) the older group included individuals who were 60 years and older.

### 2.3. Statistical Analysis

The sample size of this study was based on power analysis (*α* = 0.05, *β* = 0.8), and all the parametric variables (e.g., pedicle width and height) were checked for normal distribution. Statistical analysis was done via SPSS version 20. The Student *t*-test was used for each gender separately to compare the studied groups (control vs. stenosis) for all the parametric variables and to examine the association between pedicle diameters and age. Pearson correlation and one-way ANOVA were also used to determine the association between pedicle parameters and BMI and lumbar levels. A logistic regression analysis via the “Forward LR” method was used (separated by gender) to define the association between DLSS and pedicle parameters (dependent variable: DLSS; independent variables: pedicle width, pedicle height, age, and BMI). The intraclass correlation (ICC) coefficients were calculated to determine the intratester and intertester reliability of the measurement taken (repeated measurements of 20 individuals). Intratester reliability of the measurements was assessed by one of the authors (JA) who took the measurements twice within intervals of 3-5 days. Intertester reliability involved two testers (JA and KH) who took the measurements within an hour of each other. Both testers were blinded to the results of the measurements. Significant difference was set at *P* < 0.05.

## 3. Results

The intra- and interclass correlations for pedicle parameters ranged from 0.867 to 0.976 and from 0.751 to 0.943, respectively.

No significant differences were found in the mean age of the control males and females compared to their counterparts in the stenosis group (Table 1). However, BMI values were significantly greater in the stenosis groups compared to their counterparts in the control group.

### 3.1. Pedicle Parameters in the Study Groups

The mean PWs in both males and females in the stenosis group were significantly greater compared to those in their counterparts in the control group (Table 1). Furthermore, mean PHs for stenosis females were considerably smaller on L4 and L5 levels compared to those for their counterparts in the control group.

Our results indicate that L5 PW in both genders and L1 PW, L4 PH, and L5 PH for females are significantly associated with DLSS (Table 2).

Of the 180 individuals in the control group, 41 subjects (22.7%) had PW less than 5 mm on the upper lumbar spine (L1 and L2), compared to only 12 subjects (11.8%) in the stenosis group (Table 3). In addition, 50% to 94% of the control group had PW less than 6 and 7 mm, respectively, on the upper lumbar region, compared to 27.3% to 60.7% in the stenosis group.

### 3.2. The Association between Pedicle Parameters and Gender, Age, BMI, and Lumbar Levels

Analysis of the control group (*n* = 180) revealed that males have larger values of PW and PH than females along all the lumbar levels (*P* < 0.001). Generally, PWs and PHs were not associated with age and BMI (*P* > 0.05).

A significant increase in PWs was noted as we descend caudally (*P* < 0.001), except between L1 and L2 (*P* = 0.810): L1 = 7 mm ± 1.7, L2 = 7.3 mm ± 1.6, L3 = 8.8 mm ± 1.8, L4 = 10.7 mm ± 1.9, and L5 = 15.2 mm ± 2.2. In contrast, lumbar PH values significantly decrease caudally (*P* < 0.001) with the exception of L2 and L3 (*P* = 0.743): L1 = 14.9 mm ± 1.4, L2 = 14.1 mm ± 1.3, L3 = 14 mm ± 1.2, L4 = 13 mm ± 1.2, and L5 = 11.7 mm ± 1.2.

## 4. Discussion

The most important finding of the present study is that individuals with DLSS manifest larger pedicle width compared to the general population. This result is not surprising, as we initially assumed, because lumbar vertebral body size was greater in individuals with DLSS relative to the general population. Similarly, others have reported a significant correlation between vertebral body size and pedicle dimensions [28, 29].

It is noteworthy that pedicle parameters in the stenosis and control groups were consistently similar to the trend observed in previous studies [5, 15, 17, 30]: PW values increase caudally whereas PHs decrease from L1 to L5.

Pedicle morphometry is essential for developing and designing pedicle instrumentations since it has become a common tool for the spine surgeons. Likewise, the use of a transpedicular screw is also widespread for the DLSS population when segmental instability exists [31]. There is a consensus that PW is the most important parameter in relation to screw size fixation due to its smaller size compared with PH [7, 20, 22]. One of the main factors regarding the rigidity of the fixation system is the pullout strength of the transpedicular screw [32]. It has also been reported that an increase of 1 mm of the screw diameter improves the pullout strength [14, 33]; thus, a wider screw results in better fixation. The outer screw diameter should match precisely the internal transverse diameter of the pedicle without exceeding its external borders [19].

The outer diameters of the most commonly used pedicle screws range from 5 to 7 mm [20, 34]. The result regarding PWs in the control group showed that 22.7% of this group had PW of less than 5 in the upper lumbar spine (L1 and L2) which was quite similar to results obtained from the studies of Scoles et al. [16] and Ofiram et al. [35]. In comparison, only 0 to 2.2% of the subjects have PW less than 6 and 7 mm on the lower lumbar region (L4, L5), respectively. These findings imply that (1) utilizing a screw diameter of 6 mm in the upper lumbar spine for the general population should be avoided and (2) a screw diameter of 7 mm could be acceptable in the lower lumbar region. We believe that these findings are not surprising as they are in accordance with other studies [12, 15–17]. Weinstein and colleagues have also indirectly supported this idea when they reported a 40% failure rate when attempting insertion of a screw diameter of 7 mm in the upper lumbar spine [36]. Our result also showed that the percentage of PW less than 5 and 6 mm on the upper lumbar spine for the control group is almost 3 times greater than that for the stenosis group (22.7% vs. 7.2% and 50.5% vs. 27.3%, respectively). This indicates that for DLSS individuals, compared to the general population, screws with a larger diameter could be utilized for the entire lumbar spine.

We also postulate that subjects with spine disorder might display variant pedicle morphometry. This proposal can be partially supported by Cheung and colleagues as they reported that individuals with low back pain might manifest different pedicle morphometry than the general population [10].

Comparing our outcomes of pedicle parameters (control group) with previous radiologic studies (Table 4) revealed that PWs were, in general, similar to those obtained from the Asian population [5, 15, 17, 37] but smaller than those of Western studies [13, 20, 22, 38]. This result could emphasize the effect of differing populations on the wide disparity in the reported results [12, 15, 17]. In addition, PHs were greater than PWs and decreased caudally. The lowest value measured for PH was 8.35 mm, and the greatest value was 19.3 mm. This outcome is mostly consistent with the studies of Marchesi et al. [13] and Kadioglu et al. [39], with the exception of L5.

The current study also found that males had significantly larger pedicle diameters than females along all the lumbar levels. This result confirms what is commonly reported [5, 9, 19, 35, 38, 40] but contradicts others [10, 11]. Similar to the studies of Yu et al. [9] and Cheung et al. [10], the current study did not demonstrate any significant relationship between age and pedicle diameters. With respect to BMI, we also failed to confirm significant correlations with pedicle parameters. This result is in agreement with Ofiran et al. [35], but not with Yu et al. [9]. We assume that our finding contrasts the results of Yu and colleagues due to the differing studied populations and methods of measurements: CT images for the living population vs. direct measurement for dry specimens. It is well known that the CT scan is the gold standard for pedicle measurements [35]. Likewise, some authors found similar results for pedicle parameter values when they based their methods on both the direct measurement and CT technique [20, 22]. We believe that studies conducted on living populations are preferable to those based on cadaver specimens, because preoperative CT scans are usually used to determine the precise screw dimensions.

### 4.1. Limitation of the Study

Although this study has the largest series that was conducted on CT scans, a large-scale population with DLSS is needed to confirm the association between this phenomenon and pedicle diameters. Pedicle length and transverse pedicle angle are required to better define the screw dimensions and to avoid nerve root injury and inadvertent penetration of the screw in the spinal canal. More so, some studies have reported that the transverse pedicle angle could be smaller in subjects with degenerative spinal diseases that may be attributable to developmental changes [41, 42].

## 5. Conclusions

The current study revealed that symptomatic subjects with DLSS manifest different pedicle diameters than the general population. When planning instrumentation for DLSS individuals, the pedicles will be able to accept larger screws than those of the general population. Furthermore, pedicle diameters are gender-dependent and independent of age and BMI.

## Figures and Tables

**Table 1:** Age, BMI, PW, and PH values of the study groups (control vs. stenosis) for each gender separately.

Variables	Males			Females		
	Control (mean ± SD)	Stenosis (mean ± SD)	*P* value	Control (mean ± SD)	Stenosis (mean ± SD)	*P* value
Age (years)	62.9 ± 12.38	66.2 ± 10.82	0.066	62 ± 12.97	62.5 ± 8.63	0.795
BMI (kg/m^2^)	27.4 ± 4.21	28.9 ± 4.55	0.021	27.61 ± 5.13	31.48 ± 5.83	<0.001
L1 PW (mm)	7.7 ± 1.2	8.5 ± 2	0.005	6.3 ± 1.7	7 ± 1.3	0.007
L2 PW (mm)	8.1 ± 1.3	8.9 ± 1.7	0.001	6.4 ± 1.5	7.3 ± 1.3	<0.001
L3 PW (mm)	9.7 ± 1.6	10.7 ± 1.8	<0.001	8 ± 1.5	9.1 ± 1.5	<0.001
L4 PW (mm)	11.5 ± 1.7	12.6 ± 1.6	<0.001	9.8 ± 1.7	10.8 ± 1.4	<0.001
L5 PW (mm)	16 ± 2	17.6 ± 2.3	<0.001	14.5 ± 2	16 ± 1.9	<0.001
L1 PH (mm)	15.6 ± 1.2	15.7 ± 1.8	0.938	14.2 ± 1.2	13.9 ± 1.2	0.088
L2 PH (mm)	14.8 ± 1.1	15.1 ± 1.8	0.238	13.6 ± 1.1	13.4 ± 1.1	0.378
L3 PH (mm)	14.5 ± 1.2	14.7 ± 1.6	0.338	13.5 ± 1	13.2 ± 1.2	0.108
L4 PH (mm)	13.5 ± 1.1	13.4 ± 1.7	0.645	12.5 ± 1.1	11.8 ± 1.2	<0.001
L5 PH (mm)	12.2 ± 1.3	12 ± 1.7	0.564	11.3 ± 1	10.5 ± 1.1	<0.001

SD: standard deviation; BMI: body mass index; PW: pedicle width; PH: pedicle height.

**Table 2:** A logistic regression analysis demonstrating the variables that are significantly associated with degenerative lumbar stenosis (males and females listed separately).

	OR	95% CI	*P* value
Males			
Age	1.032	1.003-1.063	0.029
BMI	1.079	1.000-1.165	0.050
L5 PW	1.361	1.171-1.581	<0.001
Females			
BMI	1.1	1.026-1.180	0.007
L1 PW	1.444	1.108-1.883	0.007
L5 PW	1.457	1.171-1.814	0.001
L4 PH	0.593	0.391-0.899	0.014
L5 PH	0.663	0.441-0.998	0.049

OR: odds ratios: CI: confidence intervals; BMI: body mass index; PW: pedicle width; PH: pedicle height.

**Table 3:** Percentage of pedicle width less than 5, 6, and 7 mm in the studied groups at the lumbar level.

Levels	Control group (*n* = 180)			Stenosis group (*n* = 165)		
	<5 mm	<6 mm	<7 mm	<5 mm	<6 mm	<7 mm
L1	13.3	28.3	49.4	4.8	16.4	35.2
L2	9.4	22.2	43.9	2.4	10.9	25.5
L3	1.7	3.9	15.6	0	1.2	3.6
L4	0	0	2.2	0	0	0
L5	0	0	0	0	0	0

**Table 4:** Pedicle diameters in the current study compared with only radiologic studies.

Study	Mean diameters (mm)	L1	L2	L3	L4	L5
Current study (*n* = 180)	PW	7	7.3	8.8	10.7	15.2
	PH	14.9	14.1	14	13	11.7

Mohanty et al., 2018 (*n* = 102-124)	PW	7.2	7.6	8.4	10.1	13

Acharya et al., 2010 (*n* = 50)	PW	7.20	7.62	8.97	11.12	13.91

Chadha et al., 2003 (*n* = 14-20)	PW	6.69	7.26	8.43	10.81	13.47

Kadioglu et al., 2003 (*n* = 29)	PW	8.8	9.7	10.3	10.8	14.6
	PH	14.7	14.5	13.6	13.6	13.4

Mitra et al., 2002 (*n* = 20)	PW	7.34	7.45	8.51	9.71	14.49
	PH	16.42	15.65	15.24	15.29	15.17

Cheung et al., 1994 (*n* = 35-134)	PW	5.3	6.7	9.5	11.5	14.7

Bernard and Seibert, 1992 (*n* = 154)	PW	ND	8.13	8.7	10.88	14.54

Olsewski et al., 1990 (*n* = 37-42)	PW	8.2	8.3	10	12.6	16.6
	PH	18.2	17.2	16.9	15.6	13.8

Marchesi et al., 1988 (*n* = 28-46)	PW	7.1	7.8	9.7	13	18
	PH	15.4	14.8	14.2	13.9	13.7

Zindrick et al., 1987 (*n* = 26-56)	PW	8.7	8.9	10.3	12.9	18
	PH	15.4	15	14.9	14.8	14

Krag et al., 1986 (*n* = 14-24)	PW	7.01	8.67	9.30	11.03	15.15

ND: no data; PW: pedicle width; PH: pedicle height.

## Data Availability

The data used to support the findings of this study are available from the corresponding author upon request.
